# Face mask use in healthcare settings: effects on communication, cognition, listening effort and strategies for amelioration

**DOI:** 10.1186/s41235-021-00353-7

**Published:** 2022-01-10

**Authors:** Emily Lee, Kayla Cormier, Anu Sharma

**Affiliations:** Brain and Behavior Laboratory, Department of Speech Language and Hearing Sciences, University of Colorado Boulder, 2501 Kittredge Loop Drive, 409 UCB, Boulder, CO 80309-0409 USA

## Abstract

**Aim:**

To investigate mask use and the difficulties it may pose during communication in healthcare settings.

**Methods:**

A survey utilizing a series of Likert scales was administered. Mask use challenges between clinicians and their patients were examined in the domains of communication, listening effort, cognition, and rehabilitation.

**Results:**

Across 243 participants, mask use significantly increased listening effort, with hearing loss having an additive effect on listening effort. Listening effort was also significantly associated with more trouble understanding conversation, decreased interest in conversation, more difficulty connecting with patients, changes in cognition for both providers and patients, and changes in the clinical efficiency of providers. Hearing loss had an additive effect for trouble understanding conversations and changes in clinical efficiency.

**Conclusion:**

These results provide information about the clinical strain introduced from mask use in healthcare settings. Overall, results show that in healthcare settings there is increased cognitive load and listening effort for both patients and providers, as well as changes in clinical efficiency for providers when utilizing masks. These effects are often greater with hearing loss. Results showed that patients reported written and visual instructions would be most beneficial to include in appointments among the other rehabilitative strategies which are discussed.

## Significance statement

Evidence suggests that COVID-19 could be transmitted before symptom onset and community transmission might be reduced if everyone, including people who have been infected but are asymptomatic and contagious, wear face masks (Feng et al., 2020). The World Health Organization (WHO) noted that mask use in the medical care setting introduces potential harms and risks that “create difficulty communicating for persons relying on lip reading” when considering members of the Deaf and Hard of Hearing community (World Health Organization, 2020). The research described in this study investigated aspects of challenges that mask use poses between the clinician and patient and showed that mask use increases listening effort for both provider and patients, decreases clinical efficiency for providers and has negative communication consequences for patients with hearing loss. The use of a survey allowed us to examine a unique experience in a timely manner. We document and discuss communication difficulties that patients and their health care providers (including those with hearing loss) may be facing while using masks, especially in the domains of listening effort and cognition. Further we identified rehabilitative strategies which may offset some of these difficulties. Our results will provide much-needed information about clinical strains with mask use in healthcare settings and modifications that may allow an improved experience with facemasks for both patients and providers in healthcare settings.

## Background

To slow the rate of spread of COVID-19, most states in the U.S. currently either require or recommend masks to be worn statewide or have some sort of requirement in certain locales like healthcare settings. Evidence suggests that COVID-19 can be transmitted before symptom onset and community transmission might be reduced if everyone, including people who have been infected but are asymptomatic and contagious, wear face masks (Feng et al., 2020).

Wearing a face mask is common practice for certain healthcare providers in particular clinical settings, but because a vast majority of healthcare providers and patients were not required to wear masks previously, many may have experienced difficulty in communication for the first time. The World Health Organization (WHO) recognized this devastating impact when it noted that mask use in the medical care setting introduced potential harms and risks that “create difficulty in communicating for persons relying on lip reading” when considering members of the Deaf and Hard of Hearing community (World Health Organization, 2020). Previous research has shown detrimental effects of wearing a face mask in clinical contexts with limited access to facial expressions as this can influence the viewer’s emotional state, attitudes, and subsequent behaviors (Channouf, 2000) as well as patient satisfaction, medical advice, medication compliance, and positive clinical outcomes (Beck, Daughtridge, & Sloane, 2002). There are numerous negative effects that have been reported with mask use including patient concerns during medical appointments regarding noise attenuation and lip-reading, patient understanding, perceived empathy, trust, and more (Corey et al., 2020; Fröhlich et al., 2021; Kratzke et al., 2021; Rahne et al., 2021; Trecca et al., 2020).

Conventional surgical masks block visual access to the mouth and obscure other potential facial cues, creating difficulty with communication, especially for those with hearing loss. Cory et al. (2020) reported attenuation of sounds above 1000 Hz for all options of mask types. Research findings confirm improved speech perception in noise for listeners with a hearing loss when visual input is provided using a transparent surgical mask (Atcherson et al., 2017). The difficulty of mask use isn’t isolated to only those with hearing loss. A recent study that assessed differences of various mask types found that in quiet, mask type had little influence on speech intelligibility, but once in noise, intelligibility dropped for all types of face masks for young and older adult listeners (Brown et al., 2021). Previous research confirms that listening to degraded speech requires additional cognitive resources (especially for those with hearing loss) for successful comprehension (Peelle, 2018; Wingfield et al., 2015; Humes et al., 2013). As greater cognitive contributions are needed in processing degraded speech for comprehension, understanding the influence of mask use on these abilities is critical. Rahne et al. (2021) examined the effects of influence of acoustic attenuation by mask use on speech recognition thresholds and listening effort and reported that masks reduce speech perception accuracy and increase listening effort in various noise signals, even for those with normal hearing.

In this study, we examine whether mask use poses challenges in listening effort and cognition. For the purposes of this study, listening effort refers to resources or energy used by the listener needed to meet cognitive demands (Peelle, 2018). We used difficulty remembering information as a proxy for cognition in this study. These domains were selected because we theorized that mask use degrades the outgoing speech signal, resulting in effortful listening for the recipient, creating a need for activating cognitive functions like working memory and executive function to help the listener parse the incoming degraded acoustic input, and possibly depleting cognitive reserve by using considerable overall available resources just to hear (Glick & Sharma, 2017; Pichora-Fuller & Singh, 2006). Previous research confirms that hearing loss results in the dedication of greater cognitive resources for auditory processing to the detriment of other cognitive processes such as working memory (Lin et al., 2011). Further, hearing loss has been found to be associated with poor cognitive performance, incident dementia, and may contribute to cognitive decline (Dawes et al., 2015; Lin et al., 2011). While previous studies have focused mainly on the patient experience, we wanted to also examine healthcare providers and their interactions with patients as a function of mask use. Mask use could jeopardize the ability of healthcare providers to communicate with colleagues which may adversely affect the efficiency, effectiveness, equitability, and safety of therapeutic intervention services (Marler & Ditton, 2020). Studies assessing how healthcare providers are affected by mask use have the potential to change the dynamics of healthcare. The aim of this study is to document the communication difficulties that patients and their healthcare providers, both with and without hearing loss, may be facing using masks, and provide information on possible ameliorative strategies.

## Methods

### Study design

A survey was selected to obtain data as it provided timely information amidst the pandemic, allowing for a window into the state of mind of people as they are facing new challenges with communication. We examined communication difficulties posed by face mask use in clinical healthcare settings for both patients and healthcare providers with and without hearing loss. The survey was designed by audiology clinical experts (audiology students and audiologists) and reviewed by clinical audiology faculty at the University of Colorado Boulder. Clinical audiology expertise, literature review, and detailed item analyses from clinical experts ensured content validity. This survey was approved by the University of Colorado Boulder Institutional Review Board (IRB).

### Data collection

The survey was distributed on Qualtrics across the United States via social media by posting on Facebook group pages and sending emails and flyers targeting a wide variety of physicians with different clinical specialties (including ear nose and throat physicians, obstetricians, family nurse practitioners, general medical doctors, etc.). The survey was only allowed to be taken once per participant. Total testing time was typically 10 min. Hearing status was measured by self-report.

Questions in the survey were allotted to obtain demographic information from each participant. Outside of questions obtaining demographic information, remaining questions within the survey were developed to cover the domains of listening effort and cognition. Questions involved having participants answer questions with both conditions of “with mask use” and “without mask use” to ensure that the difference in the conditions were being captured. The questions used in the analysis of these domains can be seen in Table 1. It is important to note that questions 15 and 16 were combined for analysis because the same wording was used; however, question 22 and 27 were analyzed separately due to differences in wording. Other questions were specific to the participant if they reported that they were either a patient or a provider. Questions were asked in multiple ways to ensure that the participant’s responses were consistent in selecting the same response regardless of the question. Outcome variables were rated on Likert scales that allowed for one answer per question. Likert scales were not uniform, in order to more accurately capture the response options per given question. Furthermore, the survey ended with a question examining what ameliorative techniques patients would like to see implemented in healthcare settings.

**Table 1:** Survey Questions

Domain	Question	Optional Answer	Who answered it	With or Without Mask Use
Listening Effort	#14: Rate your listening effort or difficulty during appointments	1. No difficulty at all 2. A little bit 3. Moderately 4. Quite a bit 5. Extreme difficulty	Both patient and provider	With Mask use and Without Mask Use
Communication	#15: Rate your ability to understand your patients during appointments	1. No difficulty at all 2. A little bit 3. Moderately 4. Quite a bit 5. Extreme difficulty	Provider	With Mask use and Without Mask Use
Communication	#16: Rate your ability to understand your healthcare provider during appointments	1. No difficulty at all 2. A little bit 3. Moderately 4. Quite a bit 5. Extreme difficulty	Patient	With Mask use and Without Mask Use
Communication	#17: Do you have more trouble understanding people around you in a clinical setting when you are wearing a mask?	1. Never 2. Sometimes 3. About half the time 4. Most of the time 5. Always	Both patient and provider	With Mask use
Communication	#22: How would you rate your ability to connect with patients?	1. Extremely easy 2. Moderately easy 3. Slightly easy 4. Neither easy nor difficult 5. Moderately difficult 6. Extremely difficult	Provider	With mask use And Without mask use
Communication	#28: How interested are you in participating in conversations?	1. Not at all 2. A little 3. A moderate amount 4. A lot 5. A great deal	Patient	With Mask use And Without Mask Use
Clinical Efficiency	#26: Overall, rate your change in efficiency as a provider as a result of mask use	1. Not at all 2. A little bit 3. A moderate amount 4. A lot 5. A great deal	Provider	With Mask Use
Cognition	#27: Do you find it more difficult remembering information or focusing during your appointments while wearing a mask?	1. Not at all 2. A little 3. A moderate amount 4. A lot 5. A great deal	Patient	With mask use
Cognition	#21: I feel like it is more difficult to remember information from my appointment with mask use	1. Almost always 2. Often 3. A moderate amount 4. Rarely 5. Not at all	Provider	With mask use

Outcome variables were rated on Likert scales that allowed for one answer per question. The survey included a total of 28 questions. Questions were categorized to analyze the experience of the patient and provider within the domains of listening effort, communication, cognition, and clinical efficiency as grouped in this table. This table shows only questions relating to these four domains of the study that were analyzed. This table indicates whether the question was directed only towards the patient, the provider, or both. The column on the far right shows whether the question being asked is from the perspective of mask use or without mask

### Subjects

Inclusion criteria included patients who recently attended medical appointments while utilizing masks and health care providers utilizing masks who were 18 years of age or older and English speakers. Of the 296 participants (ages 19–86 years) who accessed the survey, 243 (82.8%) fully completed the survey and were included in the analysis. Participants consisted of 30 males, and 213 females. A power analysis, prior to survey collection, with power (1–*β*) set at 0.80 and α equal to 0.05 (two-tailed) indicated that a sample size of n-PA of 31 was needed to detect a small effect size (*d* = 0.2) given that we are comparing ordinary least squared linear regression models with only one difference in the number of parameters (PA-PC = 1). We had four types of respondents in our study: ‘Healthcare Providers with Hearing Loss’ (*n* = 49), ‘Healthcare Providers without Hearing Loss’ (*n* = 154), ‘Patients with Hearing Loss’ (*n* = 18), and ‘Patients without Hearing Loss’ (*n* = 22). The participants of this survey were largely female (87%). No significant differences in age between respondent types was found using a two-way analysis of variance (ANOVA) test. However, when collapsing across hearing loss results indicated significant differences in average age in healthcare providers compared to patients (*F*(3,242) = 4.24, *p* = 0.04), such that patients were older on average.

Questions were asked to gain an understanding of the masks being used by both patients and healthcare providers during medical appointments. Healthcare providers and patients were able to select the following mask types which they utilized in their medical appointments: *disposable/surgical mask*, *cloth mask*, *face shield*, *clear mask/mask with a clear panel*, *bandana*, and *none*. A majority of providers selected a *disposable/surgical mask* (47.14%), and over half of the patients indicated that their provider utilized a *disposable/surgical mask* (70.21). Providers indicated that their patients or other participants utilized a *disposable/surgical mask* the most (35.01%). Both providers and patients were asked how many medical appointments they had attended while wearing a mask which resulted in patients reporting they’ve attended a range of 1–12 medical appointments, with an average of 4.375. Providers reported that they’ve attended a range of 1–1900 medical appointments with an average of 188.504.

### Statistical analysis

Statistical analysis was performed using R studio. Data was first graphed to assess assumptions underlying linear regression analyses. Residual vs fitted graphs confirmed the linearity of our analyses. Q-Q plots were utilized to confirm assumptions of normality. Two variables (change in clinical efficiency and trouble understanding conversations) underwent log transformations to meet assumptions of normality. Ordinary least squares regression models were used to capture question responses in the domains of listening effort and communication, as well as cognition and clinical efficacy. This type of analysis allowed us to compare each variable of the model to a model without that specific variable. *P* values were adjusted to account for multiple comparisons using the Benjamini–Hochberg False Discovery Rate procedure to reduce the risk of type I errors (Benjamini & Hochberg, 1995).

## Results

### Listening effort and communication

All results were analyzed using linear regressions to examine the relationships between variables and participants’ self-reported ratings using Likert scales while controlling for age and gender. There was a main effect of change in listening effort with and without mask use (*F*(1,241) = 54.85, *p* < 0.001) across all respondents. Specifically, using a scale of 1 (no difficulty at all) to 5 (extreme difficulty), average reported listening effort without a mask was 1.21, while average reported listening effort with a mask was 2.26. Hearing loss was associated with an increase in listening effort with a mask by approximately 0.93 points on the Likert scale (*F*(1,235) = 23.87, *p* < 0.001), (as shown in Table 2). In this same regression, there was a trend towards healthcare providers demonstrating on average less listening effort than patients when utilizing masks (*F*(1,235) = 6.17, *p* = 0.061).

**Table 2:** Linear Regression Outcomes

	Beta coefficient	Delta *R*^2^	*F* value (*df*)	*P* value
Listening Effort with a Mask				
*Hearing loss*	0.93	0.09	23.87 (1,235)	< 0.001*
*Respondent*	− 0.37	0.03	6.17 (1,235)	0.061
*Age*	0.00	0.00	0.02 (1,235)	0.912
*Gender*	− 0.10	0.00	0.39 (1,235)	0.732
Respondent*hearing loss	− 0.10	0.00	0.10 (1,235)	0.890
Age*hearing loss	0.00	0.01	0.15 (1,235)	0.866
Gender*hearing loss	− 0.39	0.00	1.32 (1,235)	0.485
Trouble understanding conversation				
*Listening effort with a mask*	0.05	0.06	13.58 (1,233)	< 0.001*
*Hearing loss*	0.12	0.04	8.34 (1,233)	0.026*
*Respondent*	− 0.08	0.03	6.73 (1,233)	0.047*
*Age*	0.00	0.01	1.09 (1,233)	0.505
*Gender*	− 0.05	0.01	4.06 (1,233)	0.373
Respondent*hearing loss	− 0.09	0.01	2.15 (1,233)	0.340
Age*hearing loss	0.00	0.00	0.14 (1,233)	0.866
Gender*hearing loss	− 0.18	0.03	6.67 (1,233)	0.047*
Listening effort with a mask*hearing loss	− 0.11	0.06	15.59 (1,233)	< 0.001*
Interest in conversations with a mask				
*Listening effort with a mask*	− 0.41	0.17	6.47 (1,32)	0.063
*Hearing loss*	0.61	0.03	1.12 (1,32)	0.505
*Age*	0.02	0.11	3.83 (1,32)	0.192
*Gender*	1.01	0.10	3.45 (1,32)	0.220
Age*hearing loss	0.01	0.00	0.13 (1,32)	0.866
Gender*hearing loss	− 1.71	0.07	2.48 (1,32)	0.310
Listening effort with a mask*hearing loss	0.34	0.03	1.13 (1,32)	0.505
Connecting with patients with masks				
*Listening effort with a mask*	0.61	0.09	18.52 (1,195)	< 0.001*
*Hearing loss*	− 0.10	0.00	0.08 (1,195)	0.896
*Age*	− 0.01	0.00	0.54 (1,195)	0.651
*Gender*	0.27	0.00	0.69 (1,195)	0.588
Age*hearing loss	0.04	0.02	4.65 (1,195)	0.111
Gender*hearing loss	0.60	0.00	0.84 (1,195)	0.536
Listening effort with a mask*hearing loss	0.26	0.00	0.86 (1,195)	0.536
Cognition patient—more difficult remember				
*Listening effort with a mask*	0.45	0.19	7.50 (1,32)	0.047*
*Hearing loss*	0.13	0.00	0.05 (1,32)	0.912
*Age*	0.00	0.00	0.03 (1,32)	0.912
*Gender*	− 0.21	0.01	0.15 (1,32)	0.866
Age*hearing loss	− 0.03	0.05	1.82 (1,32)	0.384
Gender*hearing loss	0.21	0.00	0.04 (1,32)	0.912
Listening effort with a mask*hearing loss	− 0.56	0.08	2.86 (1,32)	0.260
Cognition patient—more focus				
*Listening effort with a mask*	0.44	0.33	16.08 (1,32)	< 0.001*
*Hearing loss*	− 0.41	0.03	1.10 (1,32)	0.505
*Age*	− 0.01	0.03	0.87 (1,32)	0.536
*Gender*	− 0.93	0.17	6.31 (1,32)	0.063
Age*hearing loss	− 0.02	0.10	3.37 (1,32)	0.219
Gender*hearing loss	0.99	0.05	1.78 (1,32)	0.384
Listening effort with a mask*hearing loss	− 0.37	0.08	2.88 (1,32)	0.260
Clinical efficiency				
*Listening effort with a mask*	0.51	0.14	30.37(1,195)	< 0.001*
*Hearing loss*	0.04	0.01	1.01(1,195)	0.514
*Age*	0.00	0.00	0.01 (1,195)	0.929
*Gender*	0.01	0.00	0.07 (1,195)	0.896
Age*hearing loss	0.00	0.00	0.01 (1,195)	0.929
Gender*hearing loss	− 0.03	0.00	0.18 (1,195)	0.866
Listening effort with a mask*hearing loss	0.04	0.01	1.73 (1,195)	0.384

Outcome variables were rated on Likert scales. For each of these scales, higher scores were indicative of more difficulty or greater changes with mask use, except regarding interest in conversations for which a higher score denoted more interest in conversations. Estimated beta coefficients for categorical variables denote the difference in average ratings between the groups (hearing loss vs no hearing loss, healthcare provider vs patient, female vs male). For continuous measures (age or listening effort), the beta coefficients indicate the predicted change in the outcome variable for each 1 unit increase in the predictor variable. Both trouble understanding conversations and change in clinical efficiency were log transformed for analysis. Delta R^2^ represents the percent of variable variation explained by each predictor in the model. In these linear regressions, listening effort with a mask was often associated with communication, cognitive, and clinical efficiency outcomes. All reported p-values were adjusted using the Benjamini–Hochberg False Discovery Rate procedure. *p < *0.05*

All participants were asked to rate if they were having more difficulty understanding conversations in clinical settings when wearing a mask on a scale of 1 (never) to 5 (Always). In a linear regression accounting for the potential confounds of age and gender, greater listening effort with a mask was found to be related to more difficulty understanding conversations (*F*(1,233) = 13.58, *p* < 0.001) and having hearing loss was associated more difficulty understanding conversations (*F*(1,233) = 8.34, *p* = 0.026). Additionally, there was a significant negative interaction between hearing loss and listening effort with a mask (*F*(1,233) = 15.59, *p* < 0.001), such that at the lowest level of reported listening effort, hearing loss resulted in approximately a 1.5 point increase in difficulty understanding conversation, but at the highest level of listening effort there was only about a 0.5 point increase in difficulty understanding conversation for the hearing loss group. On average, patients reported significantly more difficulty understanding conversations in a clinical setting than healthcare providers (*F*(1,233) = 6.73, *p* = 0.047). Even though males accounted for a small proportion of our sample, there was a significant additive interaction between hearing loss and gender (*F*(1,233) = 6.67, *p* = 0.047), such that males with hearing loss reported more difficulties understanding conversations than females with hearing loss.

Participants who identified as patients in this study reported their interest in having conversations on a scale of 1 (not at all) to 5 (a great deal). There was a trend towards interest in conversations being decreased as listening effort with a mask increased (*F*(1,32) = 6.47, *p* = 0.063).

Healthcare providers specified their ability to connect with their patients while utilizing masks on a Likert scale of 1 (extremely easy) to 6 (extremely difficult). A significant main effect of change in ability to connect with patients with and without a mask was noted (*F*(1,200) = 41.56, *p* < 0.001), regardless of hearing loss. Specifically, the average reported ability to connect with patients without a mask was 1.71, while the average reported ability to connect with a mask was 2.89. Listening effort with a mask was observed to be significantly related to the ability to connect with patients (*F*(1,195) = 18.52, *p* < 0.001), such that greater listening effort with a mask decreased providers' perceived ability to connect with their patients.

### Cognition and clinical efficiency

Changes in cognition with mask use were measured in patients by inquiring about increases in focusing during appointments with masks being utilized on a Likert scale of 1 (not at all) to 5 (a great deal). Greater listening effort with a mask was associated with increased focus (*F*(1,32) = 16.08, *p* < 0.001). Patients also reported increases in difficulty remembering information on a scale of 1 (not at all) to 5 (almost always). Listening effort with a mask was a significant predictor of greater difficulty in remembering information from appointments (*F*(1,32) = 7.50, *p* = 0.047).

Healthcare providers denoted changes in clinical efficiency due to mask use on a Likert scale of 1 (not at all) to 5 (a great deal). When accounting for age, gender, and hearing loss constant, listening effort with a mask was significantly related to greater changes in clinical efficiency (*F*(1,195) = 30.37, *p* < 0.001), such that as listening effort increased 1 point on the Likert scale average changes in clinical efficiency increased by approximately 0.5 of a point (Table 2).

### Strategies for rehabilitation

**Fig. 1:**
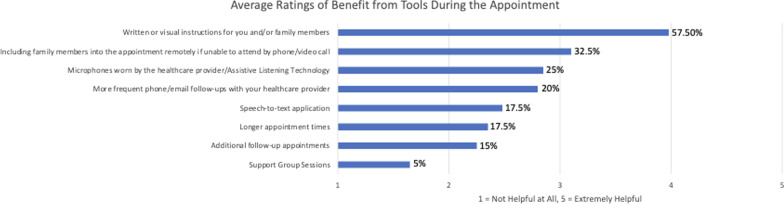
Rehabilitative Strategies. Outcome variables were rated on Likert scales of 1 (not helpful at all) to 5 (extremely helpful). For each of these scales, higher scores were indicative of the more beneficial the given rehabilitative strategy would be to have during a medical appointment

To better understand how patients feel medical appointments could be improved, patients rated the following eight rehabilitative strategies from least helpful to what is most helpful during clinical appointments, which can be seen in Fig. 1. A majority (57.5%) of patients selected *written or visual instruction for themselves and/or their family members* to be the most beneficial to have during their medical appointments, followed by *including a family member into the appointment remotely if unable to attend by phone/video call* (32.5%), *microphones worn by the healthcare provider/Assistive listening technology* (25%), *more frequency phone/email follow-ups with your healthcare provider* (20%), *speech-to-text application* (17.5%), *longer appointment times* (17.5%), *additional follow-up appointments* (15%), and *support groups* (5%). The strategies for rehabilitation were generated from the input of clinical audiologists and graduate audiology students rotating through various clinical settings. These patient views on these potential strategies provide insight on what healthcare providers may implement to improve the patient experience during their appointments.

## Discussion

Our results cast a new light on concerns regarding mask use and their implications for communication in clinical settings between healthcare providers and patients. The findings of this study describe that mask use creates an increase in listening effort, difficulty in communication, increased cognitive load, and slows clinical efficiency in healthcare settings and more so for persons with hearing loss.

Our study highlights the implications of increased listening effort with mask use. First, mask use increased listening effort overall, which is consistent with previous research that reported in daily life, the sight of a speaker who uses a face mask has the potential to increase the subjective perceived listening effort in normal hearing adults (Rahne et al., 2021). Second, we found that hearing loss was significantly associated with increased rated listening effort with mask use overall. This finding is consistent with previous research that shows that effortful listening is commonly reported in individuals with hearing loss (Pichora-Fuller, 2016). Given that conversations between providers and patients are the cornerstone of health care dynamics, the negative relationship between mask use, interest in conversations, and listening effort is particularly worrisome. Individuals with hearing loss are already at greater risk of social withdrawal and isolation (Mick et al., 2014), therefore, this observation reinforces the need for providing patients with alternatives and resources to ensure that mask use does not create another barrier that prevents them from remaining socially engaged, especially regarding their healthcare. Lastly, listening effort with a mask was a significant predictor of greater difficulty in remembering information from appointments. This suggests that added listening effort likely increased cognitive load causing difficulty retaining information from clinical appointments. Kratzke et al. (2021) also showed that mask use negatively affects patient understanding, perceived empathy, and trust due to an inability to see the surgeon's face. It has been recognized that listening can be effortful in situations that require the intensive use of cognitive processing resources as individuals may recruit extra cognitive resources to pursue specific goals as they perform listening tasks with degraded auditory input (Peelle, 2008; Lemke et al., 2016).

In terms of clinical outcomes, listening effort with mask use by the provider was also reported to significantly predict ability for providers to connect with their patients. This may in part be due to the finding that an essential part of being a good provider is listening (Brown-Johnson, et al., 2019). Additionally, as listening effort increased, clinical efficiency decreased. These findings underscore the importance of listening effort experienced by the healthcare provider and subsequent clinical care. This finding is consistent with the work of Marler and Ditton (2020) who reported that mask use adversely affects the efficiency, effectiveness, equitability and, most notably, safety of therapeutic intervention during the communication of healthcare staff with patients. This change in clinical efficiency could translate into longer time being needed for appointments and/or making more mistakes, which are both not optimal in the healthcare setting for the provider. Future research should examine the functional impacts of change in clinical efficiency due to mask use. Given the need for mask use to continue into the near future, it is important that healthcare providers find ways to compensate for some of their listening effort when they wear masks.

In a qualitative section of this study, patients identified rehabilitative strategies that could prove helpful in offsetting the challenges that accompany mask use during communication with their providers in a clinical setting. Patients rated the following eight rehabilitative strategies from least helpful to what is most helpful during clinical appointments, which can be seen in Fig. 1. The top three selected strategies that patients reported would benefit them during their medical appointments included: *written or visual instruction for themselves and/or their family members*, *including a family member into the appointment remotely if unable to attend by phone/video call*, and *microphones worn by the healthcare provider/Assistive listening technology*. Research by Cory, Jones, and Singer (2020) also suggests that active listening systems may be effective for verbal communication with masks. These results provided much-needed information about clinical modifications that may allow an improved experience with facemasks for both patients and providers in general healthcare settings. As it seems mask use will continue to persist in clinical facilities, implementing strategies such as these may improve clinical encounters and interactions needed to continue to provide quality healthcare services.

It is important to note that while these findings are novel and timely, there were limitations with the study. Many questions included in the study utilized Likert-scales and although they provide a convenient way to measure unobservable constructs, they also have the potential response bias due to a lack of objectivity (Jebb et al., 2021). Likert-scales provide limited choice options and are very susceptible to individual stylistic reporting as participants tend to agree to the statements they are shown, a phenomenon called acquiescence bias (Chyung et al., 2018). Self-report bias is another major limitation of this study as all items in this survey relied on self-reporting from participants. We relied on self-reported hearing loss which likely led to many people under or over reporting their hearing loss as it has been found that younger participants tend to overestimate and older participants underestimate their hearing loss (Kamil et al., 2015). Another caution is that this study is correlational; therefore, causation cannot be determined. Lastly, the participants included in this study are a convenience sample which explains the under-representation of the patient population and lack of diversity in gender, and the over-representation of healthcare providers and females.

Overall, our study provided a valuable perspective on communication difficulties with mask use from both an understudied population of healthcare providers, and patients. Future research should examine the best possible practices that can ease the effects of this increased listening effort in clinical settings. Understanding the struggles of both the provider and patient will shine light on the necessary modifications that will allow for not only a more efficient clinical experience, but also a higher quality of care.

## Data Availability

The datasets used and/or analyzed during the current study are available from the corresponding author on reasonable request.
